# Prognostic value of coronary computed tomography angiography in diabetic patients without chest pain syndrome

**DOI:** 10.1007/s12350-015-0213-5

**Published:** 2015-07-09

**Authors:** Inge J. van den Hoogen, Michiel A. de Graaf, Cornelis J. Roos, Aukelien C. Leen, Aan V. Kharagjitsingh, Ron Wolterbeek, Lucia J. Kroft, J. Wouter Jukema, Jeroen J. Bax, Arthur J. Scholte

**Affiliations:** Department of Cardiology, Leiden University Medical Center, Albinusdreef 2, Postal zone 2300 RC, 2333 ZA Leiden, The Netherlands; The Interuniversity Cardiology Institute of the Netherlands, Utrecht, The Netherlands; Department of Internal Medicine, Westeinde Hospital, The Hague, The Netherlands; Department of Medical Statistics and Bio-informatics, Leiden University Medical Center, Leiden, The Netherlands; Department of Radiology, Leiden University Medical Center, Leiden, The Netherlands

**Keywords:** Computed tomography (CT), diagnostic and prognostic application, diabetes, atherosclerosis

## Abstract

**Aims:**

Diabetic patients with coronary artery disease (CAD) are often free of chest pain syndrome. A useful modality for non-invasive assessment of CAD is coronary computed tomography angiography (CTA). However, the prognostic value of CAD on coronary CTA in diabetic patients without chest pain syndrome is relatively unknown. Therefore, the aim was to investigate the long-term prognostic value of coronary CTA in a large population diabetic patients without chest pain syndrome.

**Methods:**

Between 2005 and 2013, 525 diabetic patients without chest pain syndrome were prospectively included to undergo coronary artery calcium (CAC)-scoring followed by coronary CTA. During follow-up, the composite endpoint of all-cause mortality, non-fatal myocardial infarction (MI), and late revascularization (>90 days) was registered.

**Results:**

In total, CAC-scoring was performed in 410 patients and coronary CTA in 444 patients (431 interpretable). After median follow-up of 5.0 (IQR 2.7-6.5) years, the composite endpoint occurred in 65 (14%) patients. Coronary CTA demonstrated a high prevalence of CAD (85%), mostly non-obstructive CAD (51%). Furthermore, patients with a normal CTA had an excellent prognosis (event-rate 3%). An incremental increase in event-rate was observed with increasing CAC-risk category or coronary stenosis severity. Finally, obstructive (50-70%) or severe CAD (>70%) was independently predictive of events (HR 11.10 [2.52;48.79] (*P* = .001), HR 15.16 [3.01;76.36] (*P* = .001)). Obstructive (50-70%) or severe CAD (>70%) provided increased value over baseline risk factors.

**Conclusion:**

Coronary CTA provided prognostic value in diabetic patients without chest pain syndrome. Most importantly, the prognosis of patients with a normal CTA was excellent.

## Introduction

Diabetes mellitus (DM) is a major and rapidly growing global health problem. In 2013, DM was responsible for 8.4% of all-cause mortality in patients between 20 and 79 years old and 10.8% of total health expenditure worldwide.^1^ Cardiovascular complications are the leading cause of mortality in diabetic patients.^2^ Accordingly, the European Society of Cardiology (ESC) classifies patients with DM as high risk for coronary artery disease (CAD).^3^ However, not all patients with DM have CAD and also diabetic patients with CAD are often free of chest pain syndrome.^4^ Coronary computed tomography angiography (CTA) is a useful modality for non-invasive assessment of CAD. Indeed, in diabetic patients without chest pain syndrome, a high prevalence of CAD is present on coronary CTA.^5^-^7^ Potentially CTA could be used to risk stratify DM patients. However, the prognostic value of CAD on coronary CTA in these patients is relatively unknown. As a consequence, the value of coronary CTA for risk stratification of these patients is unestablished.^8^,^9^ Therefore, the aim of this study is to investigate the long-term prognostic value of coronary CTA in a large population of diabetic patients without chest pain syndrome.

## Methods

### Patients

The study population consisted of 525 diabetic patients without chest pain syndrome, referred from an outpatient diabetic clinic for assessment of cardiovascular risk between May 2005 and August 2013. The cardiovascular assessment includes coronary artery calcium (CAC) score and CTA to evaluate the presence and severity of CAD.^5^,^10^ After enrolment in the prospective clinical registry, patients underwent a non-contrast CT for CAC-scoring followed by a contrast coronary CTA. Inclusion criteria consisted of confirmed diagnosis of DM type 1 or 2 (fasting plasma glucose level ≥126 mg/dL, use of oral glucose lowering medication or insulin) and the absence of chest pain syndrome.^11^ Exclusion criteria were known or suspected (CAD), previous coronary revascularization, cardiac arrhythmias, pregnancy, and contraindications for the use of iodinated contrast media.

Clinical data were prospectively entered into the departmental Cardiology Information System (EPD-Vision©, Leiden University Medical Center, the Netherlands) and retrospectively analyzed. The Institutional Review Board of the Leiden University Medical Center approved this evaluation of clinically collected data, and waived the need for written informed consent.

### Coronary CTA Acquisition

Patients were scanned using a 64-slice or 320-row multidetector scanner (64-slice: Aquillon 64, Toshiba Medical Systems, Otawara, Japan; 320-row: Aquillon ONE, Toshiba Medical System, Otawara, Japan). Scan-protocol was followed as previously described.^12^,^13^ Post-processing of scans was performed with application of dedicated software (Vitrea FX 1.0, Vital Images, Minnetonka, MN, USA). Uninterpretable scans were excluded from the analysis.

### CAC-Scoring

CAC-scoring was performed according to the algorithm of Agatston. CAC-score was stratified into four risk categories: 0, 1-99, 100-399, ≥400.^12^

### Coronary CTA

All coronary CTAs were analyzed by consensus of experienced observers according to the modified 17 segments American Heart Association (AHA) classification.

First, each segment was assessed for interpretability. Segments were defined as uninterpretable in case of severe motion artefacts or low-contrast resolution. Additionally, segments with a diameter ≤1.5 mm were excluded.^5^ Second, interpretable segments were evaluated for stenosis. Stenosis was stratified into four categories: normal if no plaques were present on CTA, non-obstructive if the plaque covered <50%, obstructive if the plaque covered 50-70%, severe if the plaque covered >70% of the coronary artery lumen. If plaque was present, plaque composition was determined (calcified, mixed, and non-calcified). One type of plaque compositions was assigned per segment.

### Follow-Up

Follow-up data were retrospectively gathered by review of electronic medical records; blinded from CTA results, between December 2013 and February 2014, both the medical records of the department of cardiology and of the referring outpatient diabetic clinic have been analyzed. Three endpoints were registered: all-cause mortality, non-fatal myocardial infarction (MI), late revascularization. Non-fatal MI was defined based on criteria of typical chest pain, elevated cardiac enzyme levels, and typical changes on the ECG.^14^ Late revascularization was defined as percutaneous coronary intervention (PCI) or coronary artery bypass grafting (CABG) after 90 days of scan acquisition.^15^ All revascularization procedures within 90 days were considered coronary CTA-driven. For the analysis, a composite endpoint was constructed of all three endpoints.

### Statistical Analysis

All continuous data (normally distributed, non-normally distributed) are presented as mean ± SD for reasons of uniformity. Categorical data are presented as absolute numbers and percentages.

First, baseline characteristics were compared between patients with and without obstructive CAD (≥50%), similarly between patients with and without events. Second, results of both CAC-scoring and coronary CTA were compared between patients with and without events. Third, survival analyses were performed by the Kaplan-Meier method. Cumulative event-rates for CAC-score and coronary stenosis were obtained by this method, using the composite endpoint. Note that these survival analyses were crude, because no corrections for baseline characteristics were performed. Fourth, the independent prognostic value of baseline characteristics, CAC-scoring, and coronary CTA was assessed. For this purpose, univariate and multivariate Cox-regression analyses were performed. To avoid over fitting of the model, a selection of univariate significant variables was entered into the multivariate model.

All statistical tests were two-sided. Comparisons between groups were performed with the Independent-Samples *T* test or Mann-Whitney U test for continuous data and the *χ*^2^ test for categorical data. Comparisons of Kaplan-Meier curves were performed with the Log-Rank test. To compare the model fit of the multivariate Cox-regression models for CTA and CAC-score the −2 Log Likelihood was used. However, it should be noted that for non-nested models (i.e., Model 2 vs. Model 4), this only provides a crude comparison for which no *P* values could be calculated. All statistical analyses were performed with SPSS software (Version 22.0, SPSS IBM Corp., Armonk, New York). A *P* value <.05 was considered statistically significant.

## Results

### Patients

**Figure 1:**
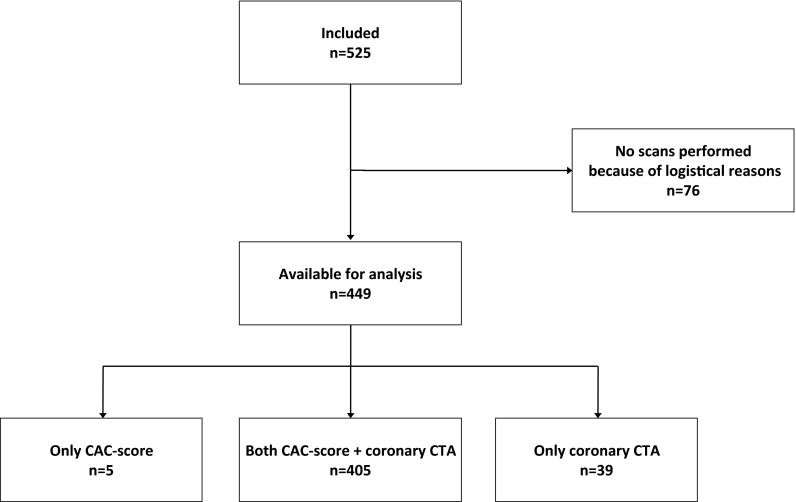
Flowchart of the study population

The study population consisted of 525 patients. As depicted in Figure 1, 76 (14%) patients were excluded from this analysis because of logistical reasons (i.e., patients who did not attend appointment). The results of 449 patients were available for the present analysis: 405 patients underwent both CAC-scoring and coronary CTA; 5 patients underwent only CAC-scoring; 39 patients underwent only coronary CTA. In total, CAC-scoring was performed in 410 patients and coronary CTA in 444 patients. Mean age was 54 ± 11 years; 265 (59%) patients were male, and median DM duration was 12 (IQR 6-22) years. Baseline characteristics of the population are depicted in Table 1.

**Table 1:** Baseline characteristics stratified according to coronary CTA results and events

Baseline	Total (n = 449)	Obstructive CAD (≥50%)			Events		
		Yes (n = 147)	No (n = 284)	*P* value	Yes (n = 65)	No (n = 384)	*P* value
Age (years)	54 ± 11	60 ± 9	50 ± 11	**<.001**	59 ± 10	53 ± 11	**<.001**
Male n (%)	265 (59%)	101 (69%)	154 (54%)	**.004**	47 (72%)	218 (57%)	**.018**
BMI (kg/m^2^)	28.6 ± 5.7	28.5 ± 5.0	28.5 ± 6.0	.942	28.7 ± 5.3	28.6 ± 5.8	.912
Hypertension^†^ n (%)	145 (33%)	65 (44%)	72 (26%)	**<.001**	28 (43%)	117 (31%)	.051
Hypercholesterolemia^‡^ n (%)	162 (36%)	68 (46%)	85 (30%)	**.001**	33 (51%)	129 (34%)	**.009**
Family history of CAD* n (%)	190 (43%)	60 (41%)	125 (45%)	.448	29 (45%)	161 (42%)	.735
Smoker n (%)	101 (23%)	41 (28%)	59 (21%)	.114	23 (35%)	78 (21%)	**.008**
DM-related risk factors							
DM type 2 n (%)	312 (70%)	111 (76%)	185 (65%)	**.028**	51 (79%)	261 (68%)	.089
DM duration (years)	15 ± 13 12 (IQR 6–22)	18 ± 14 15 (IQR 9–24)	14 ± 12 10 (IQR 5–20)	**<.001**	17 ± 14 14 (IQR 8–22)	15 ± 12 12 (IQR 5–22)	.240
HbA_1_C							
NGSP (%)	7.8 ± 1.5	7.9 ± 1.6	7.7 ± 1.5	.211	8.0 ± 1.7	7.7 ± 1.5	.287
IFCC (mmol/mol)	62 ± 16	63 ± 18	61 ± 16	.211	64 ± 19	61 ± 16	.287
Serum creatinine	78 ± 19	82 ± 19	76 ± 19	**.002**	83 ± 21	77 ± 18	**.012**
eGFR (MDRD)	76 ± 22	70 ± 20	79 ± 22	**<.001**	69 ± 21	77 ± 22	**.006**
DM-related complications				**<.001**			**.002**
PVD n (%)	20 (5%)	12 (8%)	7 (3%)		7 (11%)	13 (3%)	
PNP n (%)	97 (22%)	41 (28%)	50 (18%)		20 (31%)	77 (20%)	
PVD and PNP n (%)	26 (6%)	14 (10%)	12 (4%)		6 (9%)	20 (5%)	
DM-related treatment				.578			.083
Oral	131 (29%)	47 (32%)	79 (28%)		21 (32%)	110 (29%)	
Insulin	170 (38%)	50 (34%)	116 (41%)		18 (28%)	152 (40%)	
Oral and insulin	99 (22%)	33 (22%)	58 (20%)		21 (32%)	78 (20%)	
Medication							
Aspirin n (%)	99 (22%)	47 (32%)	46 (16%)	**<.001**	27 (42%)	72 (19%)	**<.001**
ACE-inhibitors n (%)	155 (35%)	73 (50%)	73 (26%)	**<.001**	34 (52%)	121 (32%)	**.001**
ARB n (%)	36 (8%)	10 (7%)	25 (9%)	.464	6 (9%)	30 (8%)	.715
Statins n (%)	248 (56%)	97 (66%)	138 (49%)	**.001**	45 (69%)	203 (53%)	**.018**
Beta-blockers n (%)	41 (9%)	20 (14%)	19 (7%)	**.018**	11 (17%)	30 (8%)	**.018**
Calcium-antagonists n (%)	14 (3%)	9 (6%)	5 (2%)	**.015**	6 (9%)	8 (2%)	**.002**
Serum markers							
Total cholesterol (mmol/l)	4.7 ± 1.1	4.6 ± 1.2	4.7 ± 1.0	.183	4.9 ± 1.2	4.6 ± 1.0	.051
LDL (mmol/l)	2.9 ± 1.0	2.8 ± 1.1	2.9 ± 1.0	.503	3.0 ± 1.1	2.8 ± 1.0	.176
HDL (mmol/l)	1.4 ± 0.5	1.4 ± 0.5	1.5 ± 0.5	.062	1.4 ± 0.5	1.4 ± 0.5	.729
Cholesterol/HDL ratio	3.6 ± 1.5	3.7 ± 1.4	3.6 ± 1.6	.594	3.8 ± 1.3	3.6 ± 1.5	.310
Triglycerides (mmol/l)	1.7 ± 1.2	1.7 ± 1.1	1.6 ± 1.2	.462	1.8 ± 1.2	1.6 ± 1.1	.262

Bold values are statistically significant (*P* < 0.05)

*ACE*, Angiotensin converting enzyme; *ARB*, angiotensin receptor blocker; *BMI*, body mass index; *CAD*, coronary artery disease; *DM*, diabetes mellitus; *eGFR*, estimated glomerular filtration rate; *HDL*, high density lipoprotein; *IFCC*, International Federation of Clinical Chemistry; *LDL*, low density lipoprotein; *MDRD*, Modification of Diet in Renal Disease; *NGSP*, National Glycohemoglobin Standardization Program; *PNP*, polyneuropathy; *PVD*, peripheral vessel disease

^† ^Blood pressure ≥140/90 mmHg or treatment with antihypertensive medication; ^‡^ total cholesterol level >5.0 mmol/L or use of cholesterol lowering medication; * presence of coronary artery disease in first-degree family members at age <55 years in men and <65 years in women

### Events

The composite endpoint of all-cause mortality, non-fatal MI, and late revascularization occurred in 65 (14%) patients. All-cause mortality occurred in 13 (3%) patients and late revascularization in 52 (12%) patients (PCI: 30 patients, CABG: 22 patients). Of the 52 patients who underwent revascularization, 27 (52%) patients were referred to invasive coronary angiography because of new-onset angina, 20 (38%) patients had documented ischemia on a single positron emission tomography (SPECT) myocardial perfusion imaging (MPI) performed after CTA, and 5 (10%) patients presented with new ischemia on SPECT MPI during follow-up. Non-fatal MI did not occur. Early revascularization, which was excluded from the composite endpoint, occurred in 16 (4%) patients (PCI: 13 patients, CABG: 3 patients). Median follow-up was 5.0 (IQR 2.7-6.5) years. No patients were lost to follow-up.

Patients with events demonstrated a higher mean age [59 ± 10 vs. 53 ± 11 (*P* < .001)] and number of males [47 (72%) vs. 218 (57%) (*P* = .018)] compared to patients without events. Furthermore, hypercholesterolemia [33 (51%) vs. 129 (34%) (*P* = .009)], smoker [23 (35%) vs. 78 (21%) (*P* = .008)], and overall DM-related complications (*P* = .002) were more frequently observed in this group (Table 1).

### CAC-Scoring

CAC-scoring was performed in 410 patients. Median CAC-score was 29 (IQR 0-294). The distribution within the CAC-risk categories was as follows: CAC-score = 0 in 144 (35%) patients, CAC-score = 1-99 in 106 (26%) patients, CAC-score = 100-399 in 67 (16%) patients, CAC-score ≥ 400 in 93 (23%) patients. In total, CAC-score = 0 and CAC-score ≥ 1 were observed in respectively 35% and 65% of patients (Table 2).

**Table 2:** Results of CAC-scoring and coronary CTA stratified according to events

CAC-scoring	Total (n = 410)	Events		
		Yes (n = 59)	No (n = 351)	*P* value
CAC-score	355 ± 800 29 (IQR 0–294)	1043 ± 1449 543 (IQR 141–1310)	239 ± 554 13 (IQR 0–177)	**<.001**
CAC-risk category				**<.001**
CAC-score = 0	144 (35%)	5 (9%)	139 (40%)	
CAC-score = 1–99	106 (26%)	7 (12%)	99 (28%)	
CAC-score = 100–399	67 (16%)	12 (20%)	55 (16%)	
CAC-score ≥400	93 (23%)	35 (59%)	58 (17%)	

Coronary CTA	Total (n = 431)	Events		
		Yes (n = 64)	No (n = 367)	*P* value
Coronary stenosis				**<.001**
No. of patients with normal CTA n (%)	65 (15%)	2 (3%)	63 (17%)	
No. of patients with non-obstructive CAD (<50%) n (%)	219 (51%)	11 (17%)	208 (57%)	
No. of patients with obstructive CAD (50–70%) n (%)	117 (27%)	39 (61%)	78 (21%)	
No. of patients with severe CAD (>70%) n (%)	30 (7%)	12 (19%)	18 (5%)	
Coronary plaques (stenosis)				
No. of plaques	8.0 ± 5.6	9.7 ± 4.4	7.7 ± 5.7	**.002**
No. of non-obstructive lesions	7.0 ± 5.3	6.9 ± 4.1	7.1 ± 5.4	.724
No. of obstructive lesions	1.0 ± 1.9	2.8 ± 2.8	0.6 ± 1.4	<.**001**
No. of severe lesions	0.1 ± 0.5	0.3 ± 0.8	0.1 ± 0.4	**.009**
Coronary plaques (composition)				
No. of calcified lesions	1.1 ± 2.2	2.6 ± 3.5	0.8 ± 1.8	**<.001**
No. of mixed lesions	1.5 ± 2.4	3.0 ± 3.0	1.3 ± 2.1	**<.001**
No. of non-calcified lesions	0.9 ± 1.5	1.3 ± 2.2	0.8 ± 1.4	.067

Bold values are statistically significant (*P* < 0.05)

*CAC*, Coronary artery calcium; *CAD*, Coronary artery disease; *CTA*, Computed tomography coronary angiography

The results of CAC-scoring, stratified according to events, are depicted in Table 2. Patients with events demonstrated a higher median CAC-score compared to patients without events [543 (IQR 141-1310) vs. 13 (IQR 0-177) (*P* < .001)]. Moreover, patients with events were more often classified in a higher CAC-risk category (*P* < .001).

### Coronary CTA

Coronary CTA was performed in 444 patients, of which 13 were uninterpretable. The remaining results of 431 patients were used for the present analysis. A high prevalence of CAD (85%) was demonstrated on coronary CTA: non-obstructive CAD (<50%) in 219 (51%) patients, obstructive CAD (50-70%) in 117 (27%) patients, severe CAD (>70%) in 30 (7%) patients. A normal CTA was observed in 65 (15%) patients (Table 2).

The baseline characteristics, stratified according to coronary CTA results, are depicted in Table 1. Patients with obstructive CAD (≥50%) demonstrated a higher mean age [60 ± 9 vs. 50 ± 11 (*P* < .001)], number of males [101 (69%) vs. 154 (54%) (*P* = .004)], and median diabetes duration [15 (IQR 9-24) vs. 10 (IQR 5-20) (*P* < .001)) compared to patients with no or non-obstructive CAD (<50%). Furthermore, hypertension [65 (44%) vs. 72 (26%) (*P* < .001)], hypercholesterolemia [68 (46%) vs. 85 (30%) (*P* = .001)], and overall DM-related complications (*P* < .001) were more frequently observed in this group.

The results of coronary CTA, stratified according to events, are depicted in Table 2. Patients with events presented with more severe coronary stenosis compared to patients without events (*P* < .001). Moreover, a higher mean number of plaques [9.7 ± 4.4 vs. 7.7 ± 5.7 (*P* = .002)], mean number of obstructive lesions [2.8 ± 2.8 vs. 0.6 ± 1.4 (*P* < .001)], and mean number of severe lesions [0.3 ± 0.8 vs. 0.1 ± 0.4 (*P* = .009)] were observed in this group. In addition, a higher mean number of calcified lesions [2.6 ± 3.5 vs. 0.8 ± 1.8 (*P* < .001)] and mixed lesions [3.0 ± 3.0 vs. 1.3 ± 2.1 (*P* < .001)] were present in patients with events.

### Kaplan-Meier Analysis

**Figure 2:**
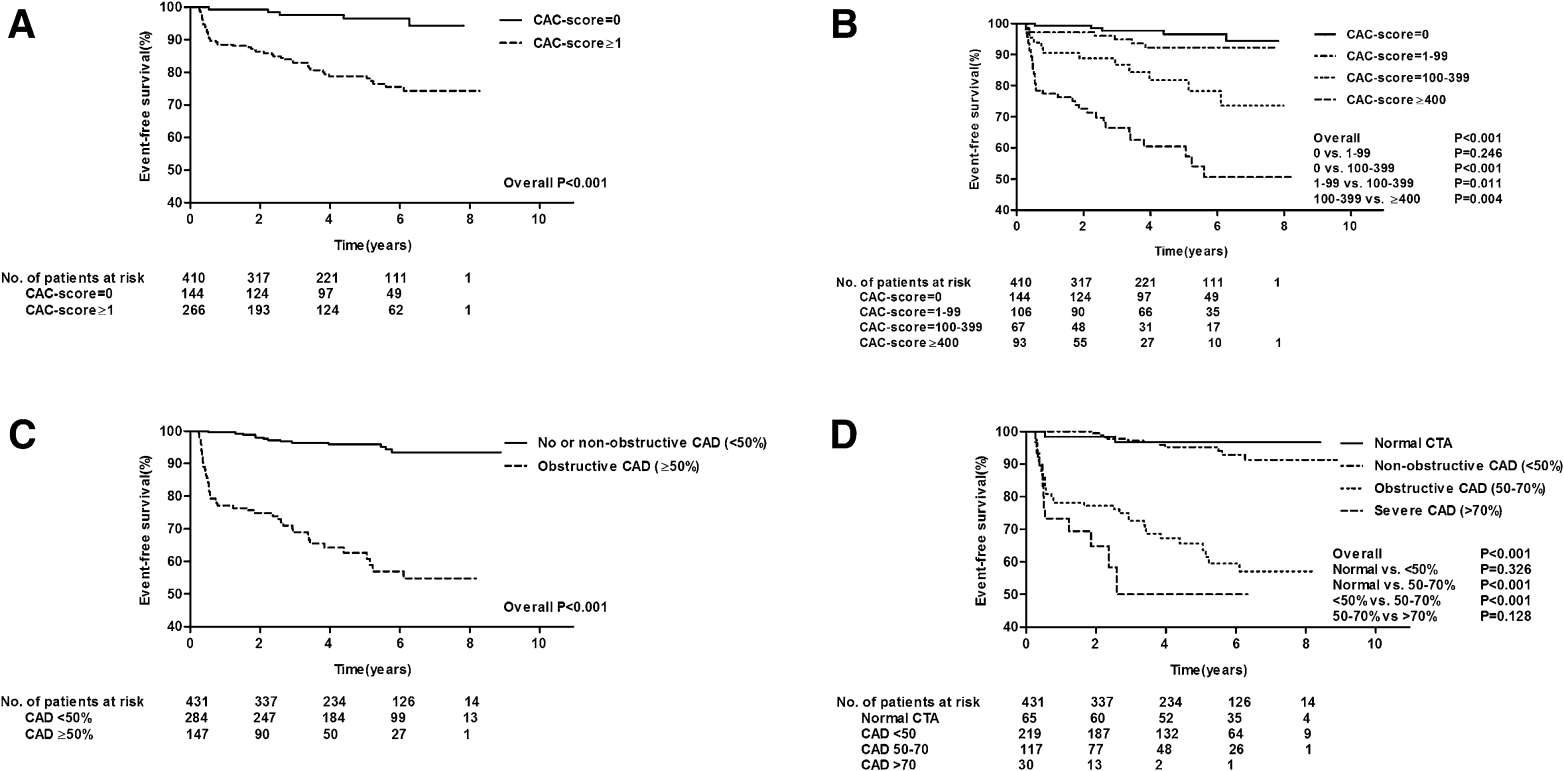
Kaplan-Meier curves for the composite endpoint (all-cause mortality, non-fatal MI, late revascularization) according to CAC-score and coronary stenosis. (**A**) Event-free survival difference between patients with CAC-score = 0 and CAC-score ≥ 1. (**B**) event-free survival difference between patients with CAC-score = 0, CAC-score = 1-99, CAC-score = 100-399 and CAC-score ≥ 400. (**C**) event-free survival difference between patients with no or non-obstructive CAD (<50%) and obstructive CAD (≥50%). (**D**) event-free survival difference between patients with normal CTA, non-obstructive CAD (<50%), obstructive CAD (50-70%) and severe CAD (>70%)

The results of the Kaplan-Meier survival analyses, stratified according to CAC-score, are depicted in Figure 2A and B. Crude event-rate was lower in patients with CAC-score = 0 compared to patients with CAC-score ≥ 1 [5/144 (3%) vs. 54/266 (20%) (*P* < .001)] (A). Additionally, an incremental increase in event-rate was observed with increasing CAC-risk category: 5/144 (3%) for CAC-score = 0, 7/106 (7%) for CAC-score = 1-99, 12/67 (18%) for CAC-score = 100-399, 35/93 (38%) for CAC-score ≥ 400 (*P* < .001). Thus, event-rate was highest in patients with CAC-score ≥ 400 (B).

The results of the Kaplan-Meier survival analyses, stratified according to coronary stenosis, are depicted in Figure 2C and D. Crude event-rate was lower in patients with no or non-obstructive CAD (<50%) compared to patients with obstructive CAD (≥50%) [13/284 (5%) vs. 51/147 (35%) (*P* < .001)] (C). An excellent prognosis was observed in patients with a normal CTA [event-rate 2/65 (3%)]. Of note, the 2 patients with a normal CTA presented with complications not related to diabetes and died of a (presumably) non-cardiac course. Additionally, an incremental increase in event-rate was observed with increasing coronary stenosis severity: 11/219 (5%) for non-obstructive CAD (<50%), 39/117 (33%) for obstructive CAD (50-70%), 12/30 (40%) for severe CAD (>70%) (*P* < .001). Event-rate was highest in patients with severe CAD (>70%) (D).

### Cox-Regression Analysis

The results of univariate Cox-regression analyses for the prediction of events are depicted in Tables 3 and 4. CAC-score ≥ 100, obstructive (50-70%) or severe CAD (>70%), total number of plaques, number of plaques stratified to stenosis (obstructive, severe) and number of plaques stratified to plaque composition (calcified, mixed, non-calcified) were all significant univariate predictors of the composite endpoint (Table 4).

**Table 3:** Univariate Cox-regression analyses of baseline characteristics for the prediction of events

Baseline	Univariate HR [95%CI]	*P* value
Age	1.06 [1.04;1.09]	**<.001**
Male	1.97 [1.14;3.38]	**.015**
BMI	1.00 [0.96;1.04]	.959
Hypertension	1.79 [1.10;2.93]	**.020**
Hypercholesterolemia	2.15 [1.32;3.51]	**.002**
Family history of CAD	0.95 [0.58;1.56]	.846
Smoker	1.98 [1.19;3.30]	**.008**
DM-related risk factors		
DM type 2	1.58 [0.87;2.85]	.132
DM duration	1.00 [1.00;1.00]	.160
HbA_1_C	1.08 [0.92;1.27]	.326
DM-related complications		
PVD	3.01 [1.37:6.60]	**.006**
PNP	1.66 [0.98;2.82]	.058
PVD and PNP	1.77 [0.76;4.10]	.184
DM-related treatment		
Oral	1.13 [0.67;1.90]	.646
Insulin	0.60 [0.35;1.04]	.069
Oral and insulin	1.79 [1.06;3.01]	**.028**
Medication		
Aspirin	2.78 [1.69;4.55]	**<.001**
ACE-inhibitors	2.39 [1.47;3.89]	**<.001**
ARB	1.03 [0.44;2.39]	.946
Statins	1.97 [1.16;3.34]	**.012**
Beta-blockers	2.29 [1.20;4.39]	**.012**
Calcium-antagonists	3.94 [1.70;9.14]	**.001**
Serum markers		
Total cholesterol	1.23 [0.99;1.54]	.067
LDL	1.13 [0.89;1.43]	.317
HDL	0.92 [0.55;1.54]	.756
Cholesterol/HDL ratio	1.06 [0.93;1.22]	.364
Triglycerides	1.09 [0.91;1.31]	.364

Bold values are statistically significant (*P* < 0.05)

Abbreviations and definitions as in Table 1

**Table 4:** Univariate Cox-regression analyses of CAC-scoring and coronary CTA for the prediction of events

	Univariate HR [95%CI]	*P* value
CAC-scoring		
*CAC-risk category*	Overall	**<.001**
CAC-score = 0	Ref. category	
CAC-score = 1–99	1.92 [0.61;6.05]	.265
CAC-score = 100–399	6.11 [2.15;17.35]	**.001**
CAC-score ≥ 400	15.79 [6.16;40.50]	**<.001**
Coronary CTA		
*Coronary stenosis*	Overall	**<.001**
Normal CTA	Ref. category	
Non-obstructive CAD (<50%)	1.92 [0.43;8.67]	.397
Obstructive CAD (50-70%)	16.18 [3.90;67.21]	**<.001**
Severe CAD (>70%)	29.03 [6.40;131.73]	**<.001**
*Coronary plaques (stenosis)*		
No. of plaques	1.09 [1.04;1.14]	**<.001**
No. of non-obstructive lesions	1.02 [0.98;1.07]	.375
No. of obstructive lesions	1.40 [1.31;1.51]	**<.001**
No. of severe lesions	2.42 [1.77;3.30]	**<.001**
*Coronary plaques (composition)*		
No. of calcified lesions	1.22 [1.14;1.30]	**<.001**
No. of mixed lesions	1.24 [1.16;1.33]	**<.001**
No. of non-calcified lesions	1.17 [1.04;1.31]	**.007**

Bold values are statistically significant (*P* < 0.05)

Abbreviations and definitions as in Table 2

The results of the multivariate Cox-regression analyses for the prediction of events are depicted in Table 5. To avoid over fitting of the model (limited number of events, n = 65), only a selection of the univariate significant variables was entered into the multivariate model (i.e., age, male gender, smoker, CAC-risk category, coronary stenosis grade). The variable smoker was selected over hypertension and hypercholesterolemia, assuming it correlated less with age and male gender. Moreover, replacing smoking with either hypertension or hypercholesterolemia did not have a strong influence on the results. In the multivariate analyses, corrected for the selected baseline variables (Model 2), CAC-score ≥ 100 was independently predictive of events. Moreover, CAC-score ≥ 400 provided incremental prognostic value over CAC-score = 100-399 [HR 12.52 (95% CI 4.29; 36.54) (*P* < .001) vs. HR 5.13 (95% CI 1.68; 15.60) (*P* = .004)]. Accordingly, obstructive (50-70%) or severe CAD (>70%) remained an independent predictor of events. The presence of severe CAD (>70%) provided incremental prognostic value over obstructive CAD (50-70%) [HR 15.16 (95% CI 3.01; 76.36) (*P* = .001) vs. HR 11.10 (95% CI 2.52; 48.79) (*P* = .001)]. Adding the CTA results to Model 2 (including baseline variables and CAC-score) resulted in a significant change in −2 Log Likelihood (17.60, *P* = .001) (Model 3). Moreover, adding the CTA results to the baseline model resulted in a larger increase in the −2 Log Likelihood compared to the adding of the CAC-score (43.78 vs. 36.30) (Model 4).

**Table 5:** Multivariate Cox-regression analyses of selected significant univariate variables for the prediction of events

Variable	Model 1		Model 2		Model 3		Model 4	
	Multivariate HR [95%CI]	*P* value	Multivariate HR [95%CI]	*P* value	Multivariate HR [95%CI]	*P* value	Multivariate HR [95%CI]	*P* value
Age	1.06 [1.04;1.09]	**<.001**	1.01 [0.98;1.04]	.541	1.00 [0.97;1.04]	.809	1.03 [1.00;1.05]	.094
Male	1.80 [1.05;3.12]	**.033**	1.33 [0.74;2.39]	.339	1.00 [0.97;1.04]	.273	1.40 [0.79;2.46]	.246
Smoker	2.23 [1.34;3.73]	**.002**	1.67 [0.97;2.88]	.065	1.40 [0.77;2.57]	.139	1.84 [1.09;3.13]	**.024**
CAC-risk category			Overall	**<.001**	Overall	.069		
CAC-score=0			Ref. category		Ref. category			
CAC-score=1-99			1.74 [0.54;5.56]	.352	1.37 [0.38;4.93]	.630		
CAC-score=100-399			5.13 [1.68;15.60]	**.004**	2.54 [0.70;9.29]	.158		
CAC-score≥400			12.52 [4.29;36.54]	**<.001**	4.06 [1.11;14.82]	**.034**		
Coronary stenosis					Overall	**.002**	Overall	**<.001**
Normal CTA					Ref. category		Ref. category	
Non-obstructive CAD (<50%)					0.98 [0.18;5.36]	.978	1.60 [0.35;7.34]	.549
Obstructive CAD (50-70%)					4.70 [0.82;26.99]	.082	11.10 [2.52;48.79]	**.001**
Severe CAD (>70%)					5.54 [0.85;36.07]	.074	15.16 [3.01;76.36]	**.001**
Change in −2 Log Likelihood			36.30	**<.001** ^±^	17.60	.001*	43.78	**<.001** ^±^

Bold values are statistically significant (*P* < 0.05)

Model 1: Baseline characteristics

Model 2: Baseline characteristics + coronary artery calcium score

Model 3: Baseline characteristics + coronary artery calcium score + coronary computed tomography coronary angiography

Model 4: Baseline characteristics + coronary computed tomography coronary angiography

Abbreviations and definitions as in Table 2

^±^Compared to Model 1

*Compared to Model 2

## Discussion

The present study assessed the long-term prognostic value of coronary CTA in a large prospective registry of diabetic patients without chest pain syndrome. Coronary CTA demonstrated high prevalence of CAD (85%), mostly non-obstructive. Most importantly, patients with a normal CTA had an excellent prognosis. Furthermore, an incremental increase in event-rate was observed with increasing coronary stenosis severity. Finally, obstructive (50-70%) or severe CAD (>70%) was independently predictive of events, with increased value over baseline risk factors (i.e., age, male gender, smoker). Moreover, the CAC-score demonstrated a similar independent predictive value for the occurrence of events. However, the model including CTA performed better than the model with CAC-score. Besides, CTA provided some additional value over the CAC-score. Nevertheless, it should be noted that this was a crude analysis and that the present study was not designed to assess the difference in performance between CAC-score and CTA.

### CAC-Score

Previous studies widely established the prevalence of CAC in diabetic patients without chest pain syndrome.^16^-^18^ The present study assessed the prognostic value of CAC by demonstrating CAC-score ≥ 100 as independent predictor of events in diabetic patients without chest pain syndrome. Prior to our study, Raggi et al investigated the prognostic value of CAC-scoring for all-cause mortality in asymptomatic individuals.^19^ In this study, 10,377 asymptomatic individuals were prospectively included to undergo electron beam computed tomography (EBCT): 903 (9%) individuals with DM, 9474 (91%) individuals without DM. This study, with mean follow-up of 5 years, demonstrated CAC as independent predictor of all-cause mortality in both diabetic and non-diabetic asymptomatic individuals. Moreover, Anand et al investigated the prognostic value of EBCT for short-term events in 510 asymptomatic patients with DM type 2.^20^ This study, with median follow-up of 2.2 years, demonstrated CAC-score ≥ 100 as independent predictor of cardiac death, MI, acute coronary syndrome (ACS), late coronary revascularization (>60 days after EBCT), and non-haemorrhagic stroke over established cardiovascular risk factors. Additionally, the PREDICT (prospective evaluation of diabetic ischemic disease by computed tomography) study investigated the prognostic value of EBCT for cardiovascular events in 589 asymptomatic patients with DM type 2.^21^ Cardiovascular events, which were defined as death due to MI or other cardiovascular causes, non-fatal MI, unstable angina, other objective evidence of CAD, and stroke, occurred in 66 (11%) patients after median follow-up of 4 years. In the multivariate analyses, CAC-score ≥101 was independently predictive of cardiovascular events. These findings were in line with the present study. Moreover, similar to the present study, incremental prognostic value was provided with increasing CAC-risk category.

### Coronary Stenosis on CTA

Several large cohort studies assessed the prevalence of CAD in the specific setting of diabetic patients without chest pain syndrome.^6^,^17^,^22^,^23^ Similar to the present study, in these studies, the majority of asymptomatic diabetic patients presented with CAD on coronary CTA (64-93%). Accordingly, non-obstructive CAD (<50%) was most frequently observed (44-64%), whereas obstructive CAD (≥50%) was less prevalent (17-29%).

Only a few studies assessed the prognostic value of coronary CTA in diabetic patients without chest pain syndrome.^15^,^22^,^24^ From the CONFIRM (coronary CT angiography evaluation for clinical outcomes: an international multicentre) registry of 27,125 patients, Min et al selected 400 asymptomatic diabetic patients who underwent coronary CTA.^15^ The prognostic value of CTA was investigated using the same composite endpoint as in the present study. Events occurred in 33 (8%) patients after mean follow-up of 2.4 ± 1.1 years: all-cause mortality in 13 (3%) patients, non-fatal MI in 8 (2%) patients, late revascularization in 12 (3%) patients. In the multivariate analyses, corrected for selected variables (i.e., age, male gender, CAC-score), maximal stenosis severity, number of vessels with obstructive CAD (≥50%), and segment stenosis score (a marker of overall atherosclerosis extent) were independently predictive of events. Indeed, obstructive (50-70%) or severe (>70%) CAD provided prognostic value in the present study. Also Faustino et al investigated the prognostic value of coronary CTA for cardiovascular events in 85 asymptomatic patients with DM type 2.^24^ Cardiovascular events occurred in 10 (11.8%) patients after median follow-up of 48 (IQR 18-68) months: cardiovascular death in 2 (2.4%) patients, unstable angina in 1 (1.2%) patients, stroke 7 (8.4%) in patients. In the multivariate analyses, corrected for univariate significant variables, the absence of obstructive CAD (≥50%) was independently protective of events. Indeed, no or non-obstructive CAD (<50%) was not associated with increased risk for events in the present study. Most importantly, patients with a normal CTA had an excellent prognosis. Last, Park et al investigated the prognostic value of coronary CTA for cardiovascular events in 557 asymptomatic Korean patients with DM type 2.^22^ Cardiovascular events were defined as cardiovascular death, non-fatal MI, ACS requiring hospitalization, and late revascularization (>6 months after coronary CTA). More cardiovascular events and lower 3 years event-free survival rates were observed in patients with obstructive CAD (≥50%) compared to patients without obstructive CAD (<50%). Accordingly, in the present study, a higher crude event-rate was observed in patients with obstructive CAD (≥50%) compared to patients with no or non-obstructive CAD (<50%).

### Coronary Plaque Composition on CTA

Multiple studies assessed plaque composition on coronary CTA in diabetic patients without chest pain syndrome. Comparable to the present study, the majority of these studies described an increased prevalence of mixed lesions in asymptomatic diabetic patients.^6^,^17^,^25^

The prognostic value of coronary plaque composition on coronary CTA for cardiovascular events in the specific setting of diabetic patients without chest pain syndrome has not been previously established. The present study demonstrated all coronary plaque compositions (calcified, mixed, non-calcified) as univariate significant predictors of events. The prognostic value of calcified and mixed lesions was highest. These findings suggest an independent association between plaque composition and events.

However, the role of coronary plaque composition remains controversial. Gaemperli et al demonstrated the prognostic value of coronary plaque composition in 220 symptomatic patients.^26^ In contrast to the present study, mixed and non-calcified plaque provided the highest predictive value for events. On the other hand, in the CONFIRM registry, including both symptomatic and asymptomatic patients with and without DM, calcified and mixed plaque provided the strongest predictive value.^27^ Further research is needed to understand the underlying pathophysiological mechanism of the different coronary plaque compositions.

### SPECT Myocardial perfusion Imaging (MPI)

The role of SPECT MPI for screening for silent ischemia in asymptomatic diabetic patients has been previously addressed.^20^,^28^ Anand et al included 510 asymptomatic diabetic patients of whom 180 patients underwent SPECT MPI.^20^ In those patients, the event-rate was significantly increased with increasing ischemic burden on SPECT MPI demonstrating the value for risk stratification. The most important study in this field was the DIAD (detection of Ischemia in Asymptomatic Diabetics) study.^28^ In this randomized controlled trial, 1123 asymptomatic diabetic participants were randomized to SPECT MPI or no screening. After a mean follow-up of 4.8 years, the incidence of cardiac events was higher in patients with significant MPI abnormalities. However, there was no significant prognostic benefit of screening.

### Clinical Implications

The present observations demonstrate the prognostic value of CTA in diabetic patients without chest pain syndrome. Recent ESC guidelines indicate patients with DM as high risk for CAD (or very high risk if ≥1 cardiovascular risk factor was present) irrespective of chest pain symptoms. Indeed, in the present study, a great majority of patients presented with CAD on coronary CTA and the presence of obstructive (50-70%) or severe CAD (>70%) was associated with an impaired prognosis. Still, CAD was ruled-out in 15% of patients based on a normal CTA. Most importantly, the prognosis of these patients was excellent.

The value of screening for CAD in high-risk diabetic patients without chest pain syndrome was recently addressed by Muhlestein et al.^29^ In this trial, 900 asymptomatic patients were randomized to CAD screening using coronary CTA or optimal medical treatment (OMT). The trial demonstrated no survival benefit from screening with coronary CTA. Therefore, this study does not support screening in all diabetic patients. Similarly, the American Diabetes Association position statement on cardiovascular disease and risk management only recommend screening using advanced cardiac testing in patients with cardiac symptoms or ECG abnormalities.^30^ However, as also demonstrated in the present study, a large proportion of the patients had CAD on CTA and coronary CTA could identify patients with excellent prognosis. This supports the need to enrich the screening population to a high-risk population who will mostly benefit from screening using coronary CTA. Especially since coronary CTA may lead to radiation exposure and may result in unnecessary invasive testings such as coronary angiography and revascularization procedures.^30^ Potentially, coronary CTA can provide a pivotal role in tailored therapy in these diabetic patients. Patients with a normal CTA have an excellent prognosis and could be treated conservatively (OMT), whereas patients with an abnormal CTA may benefit from additional non-invasive or invasive evaluation.

## Limitations

Several limitations of the present study need to be considered. First, the present study was a single-centre study. Second, the composite event-rate was relatively low. As a consequence, the study was underpowered to include all baseline risk factors into the multivariate model. Third, the endpoint mainly consisted of late revascularization; therefore, conclusions regarding hard endpoints are not justified based on this study. We cannot rule out that, despite the wide time interval, some referral bias has occurred in the patients who underwent late revascularization. Moreover, it is possible that events that have occurred in other medical centres were missed in the analysis. Fourth, coronary CTA only visualizes coronary atherosclerosis and provides no information on the hemodynamic significance of coronary stenosis.

## Conclusion

Coronary CTA provided prognostic value in a large prospective registry of diabetic patients without chest pain syndrome. Most importantly, the prognosis of patients with a normal CTA was excellent. In addition, an incremental increase in event-rate was observed with increasing CAC-risk category and coronary stenosis severity. The highest event-rate was observed in patients with severe CAD (>70%). Both CAC-score and coronary stenosis severity were independently predictive of events, after correction for baseline risk factors.

## New Knowledge Gained

Coronary CTA can be of clinical value for risk stratification of patients with diabetes mellitus without chest pain syndrome. Especially, event-free survival is excellent in patients without coronary artery disease. The prognostic value of coronary CTA seems to outweigh the prognostic value of the coronary calcium score.

## Abbreviations

CABG: Coronary artery bypass grafting
CAC: Coronary artery calcium
CAD: Coronary artery disease
CTA: Computed tomography angiography
DM: Diabetes mellitus
ESC: European Society of Cardiology
MI: Myocardial infarction
PCI: Percutaneous coronary intervention
